# Explainable incremental-value analysis of apparent diffusion coefficient and arterial spin labeling radiomics for ATRX status prediction in glioblastoma

**DOI:** 10.3389/fonc.2026.1877106

**Published:** 2026-06-19

**Authors:** Rafail C. Christodoulou, Revati Natu, Georgios Vamvouras, Platon S. Papageorgiou, Evros Vassiliou, Elena E. Solomou, Sokratis G. Papageorgiou, Michalis F. Georgiou

**Affiliations:** 1 Division of Neuroimaging and Neurointervention, Department of Radiology, Stanford University, Stanford, CA, United States; 2 Department of Information Technology, K. J. Somaiya School of Engineering, Somaiya Vidyavihar University, Mumbai, India; 3 Department of Electrical and Computer Engineering, National Technical University of Athens NTUA, Athens, Greece; 4 Department of Medicine, National and Kapodistrian University of Athens, Athens, Greece; 5 Department of Biological Sciences, Kean University, Union, NJ, United States; 6 Internal Medicine-Hematology, University of Patras Medical School, Rion, Greece; 7 1st Department of Neurology, Medical School, National and Kapodistrian University of Athens, Eginition Hospital, Athens, Greece; 8 Department of Radiology, University of Miami, Miami, FL, United States

**Keywords:** apparent diffusion coefficient, arterial spin labeling, ATRX, glioblastoma, radiomics, machine learning, explainability, MRI

## Abstract

**Introduction:**

Alpha-thalassemia/mental retardation syndrome X-linked (ATRX) mutation is an uncommon but biologically relevant molecular feature in glioblastoma (GBM), linked to tumor heterogeneity, DNA damage response pathways, and treatment-relevant biology. Noninvasive prediction of ATRX status remains challenging, and the incremental value of physiologic MRI beyond structural imaging is unclear.

**Methods:**

We analyzed 106 patients with GBM with available ATRX status and complete multiparametric MRI. Four radiomics models were compared. Model 1 used structural MRI features from contrast-enhanced T1-weighted, T2-weighted, and FLAIR images, along with age and sex. Model 1A additionally incorporated ADC and ASL-CBF radiomic features. Models 1B and 1C served as ablation models isolating the individual contribution of ADC and ASL-CBF, respectively. Six machine-learning classifiers were evaluated using stratified cross-validation, class-imbalance-aware metrics, bootstrapped confidence intervals, paired DeLong testing, and SHAP explainability.

**Results:**

The best structural model achieved an ROC-AUC of 0.721, a PR-AUC of 0.322, and a sensitivity of 0.737. Model 1A demonstrated statistically significant improvements, achieving an ROC-AUC of 0.753, a PR-AUC of 0.364, and a sensitivity of 0.947. Across classifiers, ADC and ASL improved discrimination in five of six classifiers (DeLong, p<0.05). SHAP analysis showed that age remained the dominant predictor, while ASL- and ADC-derived texture features contributed meaningful physiologic information.

**Discussion:**

ADC and ASL-CBF radiomics provide a modest but statistically significant incremental value for ATRX prediction in GBM. These findings support further validation of functional MRI sequences as a complementary radiogenomic marker.

## Graphical Abstract

**Figure:**
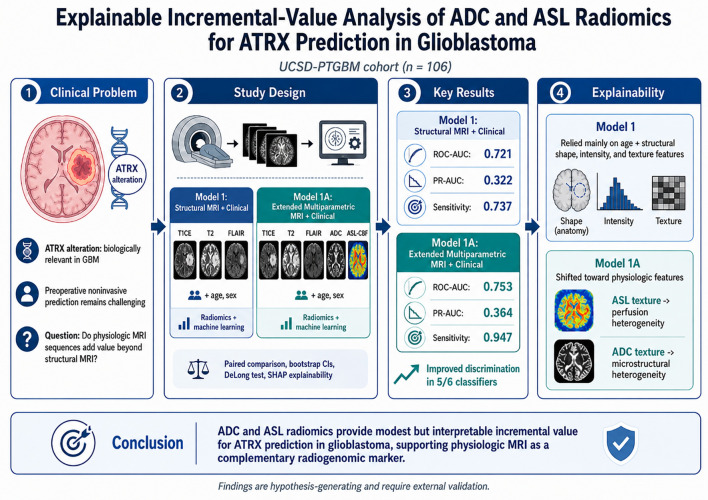

## Introduction

GBM is the most common and aggressive malignant brain tumor in adults, accounting for over 50% of cases of all adult gliomas (1). Despite recent advances in neuroimaging, surgical techniques, and treatment strategies, the overall survival rarely exceeds 15 months (2). This is largely due to its complex biology, a molecular profile that includes epigenetic and genetic abnormalities, and its heterogeneous structure (1). The 2021 WHO classification of central nervous system tumors emphasizes molecular alterations in glioma diagnosis, advancing beyond purely histological methods toward an integrated approach (3). In GBM, alterations in the ATRX gene are relatively rare but serve as important indicators of tumor heterogeneity (4). ATRX produces a chromatin-remodeling protein that plays a role in histone deposition, telomere maintenance, replication stress, and DNA damage response. Loss of ATRX has been associated with alternative telomere lengthening, genomic instability, impaired DNA repair, and disrupted cell-cycle regulation (5). These alterations often occur alongside other molecular changes, including IDH and TP53 mutations in gliomas. Studies have shown that over 75% of gliomas with these changes also harbor ATRX mutations (6). Although ATRX isn’t currently used as a standalone biomarker to guide therapy in GBM, it provides valuable insights into molecular subtypes, prognosis, and mechanisms of treatment response (7). Specifically, the interaction between ATRX loss and ATM-dependent DNA damage repair pathways suggests that ATRX status may serve as a predictive biomarker for response to DNA-damaging therapies, including temozolomide and radiotherapy, in GBM (7, 8). Therefore, ATRX status can provide crucial insights regarding GBM treatment response, molecular profiling, and patients’ survival.

ATRX status is currently determined by tissue-based immunohistochemistry or molecular testing (9). While these approaches remain the diagnostic gold standard, they involve surgical sampling and can be affected by tumor heterogeneity, sampling bias, incomplete coverage of the entire tumor, or surgery-related complications (10). A noninvasive method for estimating ATRX status from preoperative MRI could provide complementary information before surgery, support molecular risk stratification, and help guide the selection of cases for additional molecular testing (11).

Radiomics involves extracting quantitative data from MRI images, which offer valuable insights into tumor shape, intensity, and texture (12). These details help decode spatial heterogeneity, especially in highly heterogeneous tumors like GBM (1). Without such quantitative methods, these differences may be overlooked by the human eye. Recent studies indicate that MRI-based radiomics combined with machine learning can predict ATRX status in gliomas. However, this evidence is not as robust as that for IDH mutation and MGMT promoter methylation. A recent systematic review and meta-analysis showed that machine learning models can predict ATRX status from radiomic features. Nonetheless, ATRX prediction tends to have lower sensitivity than IDH prediction and is influenced by heterogeneity in tumor grade cohort selection, MRI sequences, and modeling approaches (13).

Most research on ATRX has focused on lower-grade or low- and high-grade gliomas, with only a few specifically targeting grade IV GBM (13, 14). For instance, Ren et al. (2019) analyzed ATRX loss in low-grade gliomas using radiomic features from T2/FLAIR, ADC, eADC, and ASL-CBF (15). In contrast, studies by Calabrese et al. (2022), focusing on GBM, employed broader multiparametric approaches to predict various genetic alterations, rather than assessing the added value of physiologic MRI sequences for ATRX (14).

Conventional structural MRI sequences, including contrast-enhanced T1-weighted, T2-weighted, and FLAIR imaging, detect features such as enhancement, edema, necrosis, morphology, and spatial heterogeneity. However, they offer limited direct insights into tumor cellularity and perfusion (16). This is a significant gap because ADC reflects diffusion-related microstructural heterogeneity, whereas ASL-derived cerebral blood flow captures vascular and perfusion heterogeneity (16). Sequence-level studies also indicate that T2-weighted imaging is the most informative conventional sequence for predicting ATRX status, highlighting the importance of structural heterogeneity but leaving the value of physiologic MRI unclear (17).

The added benefit of physiologic MRI for ATRX prediction in GBM is still uncertain. We hypothesized that ADC and ASL radiomic features would improve explainable prediction of ATRX status beyond structural MRI and clinical variables. To test this hypothesis, we compared a structural MRI model using T1CE, T2, and FLAIR radiomic features, along with age and sex, with an extended multiparametric model that additionally incorporated ADC and ASL-CBF features and used SHAP analysis to identify the clinical and imaging features that contributed most to prediction.

## Methods

### Reporting standards and reproducibility

This study was designed and reported in accordance with the Checklist for Evaluation of Radiomics research (CLEAR) to improve methodological transparency, reproducibility, and completeness of radiomics reporting (18). In addition, key methodological quality principles from the Methodological Radiomics Score (METRICS) were considered, including transparent cohort definition, image preprocessing, feature extraction, feature selection, model validation, class-imbalance handling, statistical comparison, and explainability (19). To further support reproducibility and open-science practices, the full code for radiomic feature extraction, machine-learning analysis, and SHAP explainability will be made publicly available at: https://github.com/Revati-N/Radiomics_ATRX_ADC_ASL.

### Dataset and patient selection

We obtained multiparametric MRI data and corresponding clinical information from the UCSD-PTGBM collection available through The Cancer Imaging Archive (TCIA) (20). The dataset comprises 243 patients diagnosed with GBM, of whom 114 had available ATRX status labels (95 Intact, 19 Loss). We excluded eight patients lacking ADC or ASL imaging data, yielding a final cohort of 106 patients used for all model comparisons. Clinical variables, including patient age and sex, were incorporated as supplementary clinical features based on prior evidence supporting their prognostic relevance in GBM (21). Cohort demographics are summarized in **Table 1**.

**Table 1:** Cohort demographics stratified by ATRX status.

Variable	ATRX intact (n = 87)	ATRX loss (n = 19)	p-value
Age, years, mean ± SD	57.5 ± 10.2	49.2 ± 22.4	0.014
Sex, n (%)			1.000
Male	61 (70.1%)	13 (68.4%)	
Female	26 (29.9%)	6 (31.6%)	

Continuous variables are reported as mean ± standard deviation and were compared using the Mann–Whitney U test. Categorical variables are reported as n (%) and were compared using Fisher’s exact test. Statistical significance was defined as p < 0.05.

### MRI sequences

**Figure 1:**
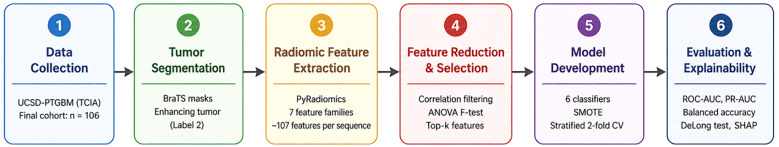
Study workflow. Overview of the radiomics pipeline for ATRX status prediction, including UCSD-PTGBM cohort selection, enhancing tumor segmentation, PyRadiomics feature extraction, correlation-based feature reduction, machine learning model development, and evaluation using ROC-AUC, PR-AUC, balanced accuracy, DeLong testing, and SHAP explainability.

We evaluated two complementary imaging configurations. Model 1 used conventional structural sequences, including contrast-enhanced T1-weighted (T1CE), T2-weighted (T2), and fluid-attenuated inversion recovery (FLAIR) imaging. Model 1A extended this configuration by incorporating diffusion-weighted ADC maps (b=4000 s/mm²) and arterial spin labeling cerebral blood flow maps (ASL-CBF), reflecting tumor cellularity and perfusion characteristics, respectively. Two additional ablation models were also evaluated: Model 1B incorporated structural MRI and ADC features only, and Model 1C incorporated structural MRI and ASL-CBF features only, to isolate the contribution of each physiologic sequence. The full study workflow is illustrated in **Figure 1**.

### Tumor segmentation

We obtained tumor segmentation masks from two sources within the UCSD-PTGBM collection on TCIA. Within this single cohort, a subset of patients underwent manual expert segmentation, while the remainder were pre-annotated using the BraTS 2024 deep learning segmentation network and subsequently refined by experts, following the same BraTS 2024 protocol and label conventions throughout. We extracted radiomic features from label 2 of the segmentation mask, corresponding to the enhancing tumor subregion in the BraTS 2024 convention, as it represents the most biologically active tumor compartment and has been shown to be relevant in prior GBM radiomic studies (22). We merged the two sources to maximize the labeled cohort, yielding 106 patients with complete multiparametric imaging and ATRX labels. Tumor segmentation masks were provided alongside the imaging volumes as part of the dataset. Segmentation masks from both data sources were generated in accordance with the BraTS 2024 protocol, ensuring consistent tumor subregion definitions and label conventions across the entire cohort.

### Radiomic feature extraction

We extracted radiomic features from each MRI sequence within the tumor mask using PyRadiomics (v3.0.1) (23). Prior to extraction, we normalized image intensities and resampled images using B-spline interpolation with a fixed bin width of 25. We extracted seven complementary feature families from the original image volumes: shape descriptors, first-order intensity statistics, grey-level co-occurrence matrix (GLCM), grey-level run-length matrix (GLRLM), grey-level size-zone matrix (GLSZM), grey-level dependence matrix (GLDM), and neighborhood grey-tone difference matrix (NGTDM). This yielded approximately 107 features per imaging sequence, resulting in approximately 321 features for Model 1, approximately 428 features for Models 1B and 1C, and 535 features for Model 1A. We appended patient age and sex to the radiomic feature vector as additional clinical predictors.

### Feature selection

**Figure 2:**
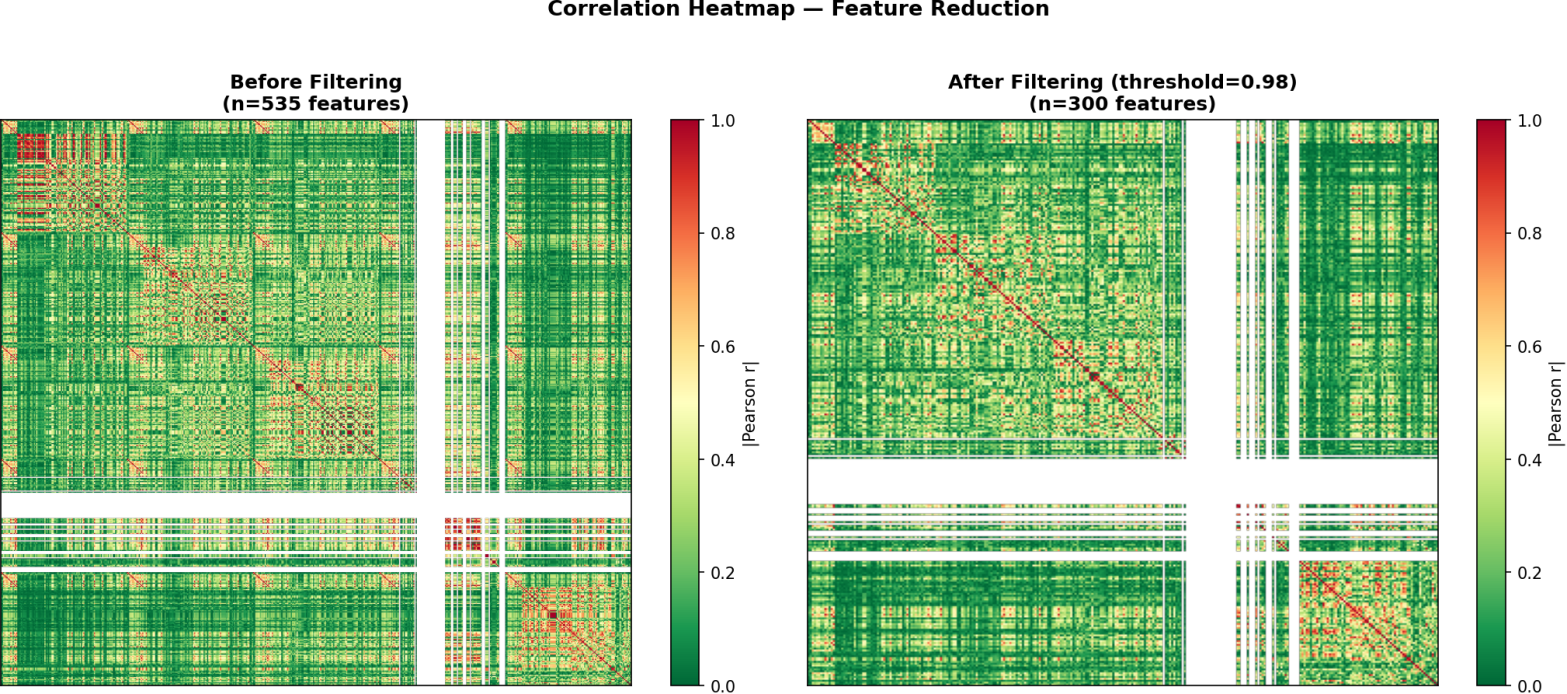
Correlation heatmap demonstrating feature redundancy reduction. Pairwise Pearson correlation heatmaps of radiomic features before and after correlation-based filtering. Before filtering, the multiparametric feature set contained 535 features and exhibited substantial inter-feature redundancy, as evidenced by clusters of highly correlated features. After applying a Pearson correlation threshold of 0.98, the feature set was reduced to 300 features, with decreased redundancy while preserving a broad distribution of structural, diffusion, and perfusion-derived radiomic information. The color scale represents the absolute value of the Pearson correlation coefficient, ranging from low correlation in green to high correlation in red.

**Figure 3:**
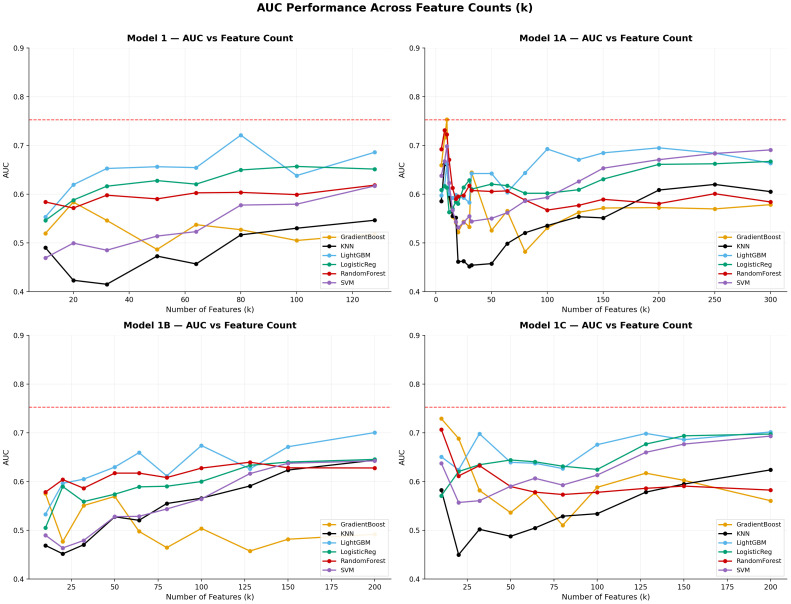
AUC performance vs feature count on each of the four models. Line plots showing ROC-AUC performance for six machine learning classifiers as top-k feature subsets increase, across all four model configurations. Model 1 included structural MRI radiomics from T1CE, T2, and FLAIR. Model 1B incorporated only structural MRI and ADC features. Model 1C incorporated only structural MRI and ASL-CBF features. Model 1A incorporated all sequences, including ADC and ASL-CBF. The dashed red line indicates the best overall performance of the multiparametric model (Model 1A, AUC = 0.753). In Model 1, LightGBM achieved the highest AUC at k = 80. In Model 1B, LightGBM achieved the highest AUC at k = 200. In Model 1C, Gradient Boosting achieved the highest AUC at k = 10. In Model 1A, Gradient Boosting achieved the highest AUC at k = 10, supporting the incremental value of combining physiologic MRI features while highlighting performance variability that is classifier- and feature-count-dependent.

To address inter-feature redundancy, we applied Pearson correlation-based filtering, removing features whose pairwise correlation exceeded a defined threshold. We determined the optimal threshold empirically by evaluating model performance across a range of values (0.80, 0.85, 0.90, 0.95, 0.97, 0.98, 0.99, and 1.0), identifying 0.95 as optimal for Model 1, 0.98 for Model 1A, and 0.97 for Models 1B and 1C. The impact of this filtering on inter-feature dependency is illustrated in **Figure 2**. We then ranked and selected features using the ANOVA F-test (SelectKBest), ordering them by their statistical separability between the ATRX Intact and Loss classes. We conducted a structured parametric analysis across progressively larger top-K feature subsets (K = 10 to 300) to evaluate performance stability and identify the optimal feature count, as shown in **Figure 3**.

### Class imbalance handling

Given the pronounced class imbalance in ATRX mutation status (95 Intact vs. 19 Loss, approximately 5:1 ratio), we applied the Synthetic Minority Oversampling Technique (SMOTE) exclusively within each training fold. SMOTE synthetically generates additional minority-class samples by interpolating between existing cases. We never applied oversampling to the test data, ensuring an unbiased performance estimate.

### Model training and evaluation

We evaluated six machine learning classifiers: Gradient Boosting, LightGBM, Logistic Regression, Random Forest, Support Vector Machine (SVM), and K-Nearest Neighbors (KNN). We trained and evaluated all models using stratified 2-fold cross-validation, which was selected to maximize the training set size given the limited cohort size and severe class imbalance. The primary evaluation metric was the area under the receiver operating characteristic curve (AUC). We systematically assessed model performance across all combinations of classifier, correlation threshold, and feature count K. Stratified 2-fold cross-validation was employed in preference to higher k configurations. Given the cohort size (n=106) and class imbalance (19 ATRX-loss cases, ~18%), a 5-fold strategy would yield approximately 3–4 minority-class samples per test fold, precluding stable performance estimation. The 2-fold strategy was therefore selected to preserve adequate minority-class representation in each test partition while maximizing the availability of training data.

### Threshold optimization and performance metrics

Given the class imbalance in ATRX status, model performance was evaluated using both threshold-independent and threshold-dependent metrics. The primary discrimination metric was ROC-AUC, with PR-AUC included as a complementary measure of minority-class performance. For operating-point analysis, the classification threshold was selected using Youden’s J statistic, defined as sensitivity + specificity − 1. Because conventional accuracy may be misleading in imbalanced cohorts, balanced accuracy, sensitivity, specificity, and PR-AUC were emphasized in model interpretation. Ninety-five percent confidence intervals were estimated using 1, 000 bootstrap iterations.

### Statistical comparison

To determine whether differences in discriminative performance among model configurations were statistically significant, we applied paired DeLong tests to all four models on the same 106-patient cohort. We defined statistical significance as p < 0.05.

### Explainability

To enhance model interpretability, we computed SHapley Additive exPlanations (SHAP) using the TreeSHAP algorithm for the best-performing classifiers. SHAP values provide an additive decomposition of each prediction, quantifying the marginal contribution of individual features to the model output. We assessed modality-level attribution by aggregating mean absolute SHAP values across features originating from the same MRI sequence, thereby identifying which imaging modalities contributed most to ATRX mutation prediction.

## Results

### Classifier-level comparison across model configurations

Across classifiers, the addition of ADC and ASL improved discrimination for five of six machine learning models. The largest gains were observed for Gradient Boosting, KNN, and Random Forest. The best structural MRI model was LightGBM with 80 selected features, achieving an AUC of 0.721, whereas the best multiparametric model was Gradient Boosting with 10 selected features, achieving an AUC of 0.753. The intermediate ablation models achieved AUCs of 0.701 (Model 1B, LightGBM, k=200) and 0.729 (Model 1C, GradientBoost, k=10), as detailed in **Table 2**.

**Table 2:** Best-performing classifier configurations for structural and multiparametric MRI radiomics models.

Model	Features	Best classifier	k	Threshold	AUC
Model 1	Structural MRI	LightGBM	80	0.95	0.721
Model 1A	Structural + ADC + ASL	GradientBoost	10	0.98	0.753
Model 1B	Structural + ADC	LightGBM	200	0.97	0.701
Model 1C	Structural + ASL	GradientBoost	10	0.97	0.729

Model 1 included radiomic features from T1CE, T2, and FLAIR. Model 1A additionally incorporated ADC and ASL-CBF features. Model 1B incorporated structural MRI and ADC features only. Model 1C incorporated structural MRI and ASL-CBF features only. For each classifier and model set, the optimal number of selected features, k, was determined using top-K feature selection.

### Model 1: structural MRI (T1CE + FLAIR + T2)

**Figure 4:**
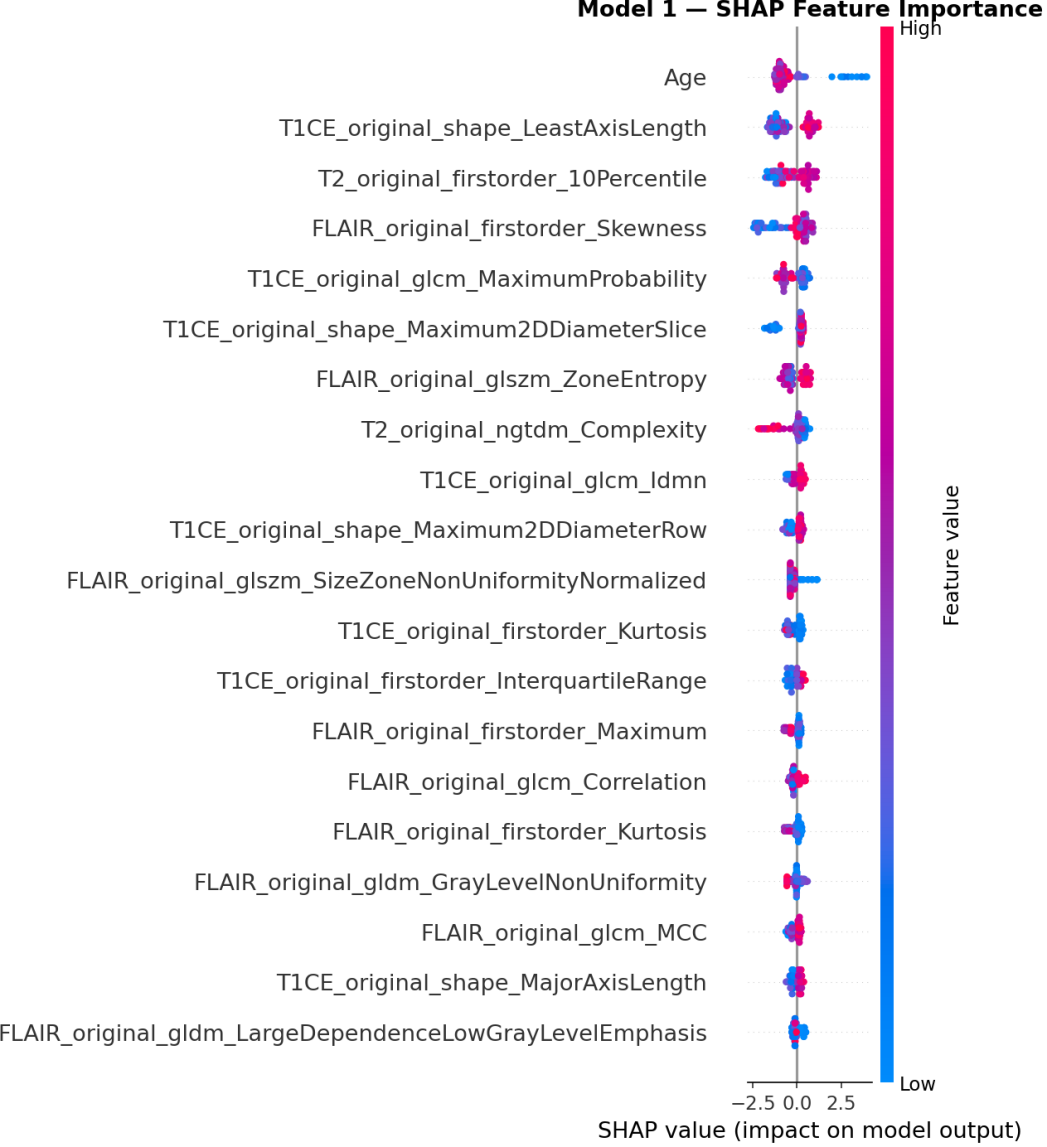
SHAP feature importance - model 1.

To evaluate whether conventional structural MRI sequences could predict ATRX mutation status, we trained and evaluated six classifiers on radiomic features extracted from T1CE, FLAIR, and T2 sequences. Radiomic extraction yielded 321 features prior to correlation filtering. Following Pearson-based redundancy reduction at a threshold of 0.95, we identified LightGBM at k = 80 features as the best-performing configuration, achieving an AUC of 0.721 (95% CI: 0.588–0.833) on the 106-patient cohort. At the Youden-derived threshold of 0.21, the model achieved a sensitivity of 0.737 (95% CI: 0.474–1.000) and specificity of 0.678 (95% CI: 0.342–0.907), with a PR-AUC of 0.322, indicating moderate discriminative ability. SHAP analysis revealed that Age was the most influential predictor, followed by T1CE-derived shape and texture features — including least axis length and GLCM_Idmn — and FLAIR-derived texture descriptors such as ZoneEntropy, indicating that tumor geometry and signal heterogeneity in structural sequences contribute meaningfully to ATRX prediction (**Figure 4**).

### Model 1A: extended multiparametric MRI (T1CE + FLAIR + T2 + ADC + ASL)

**Figure 5:**
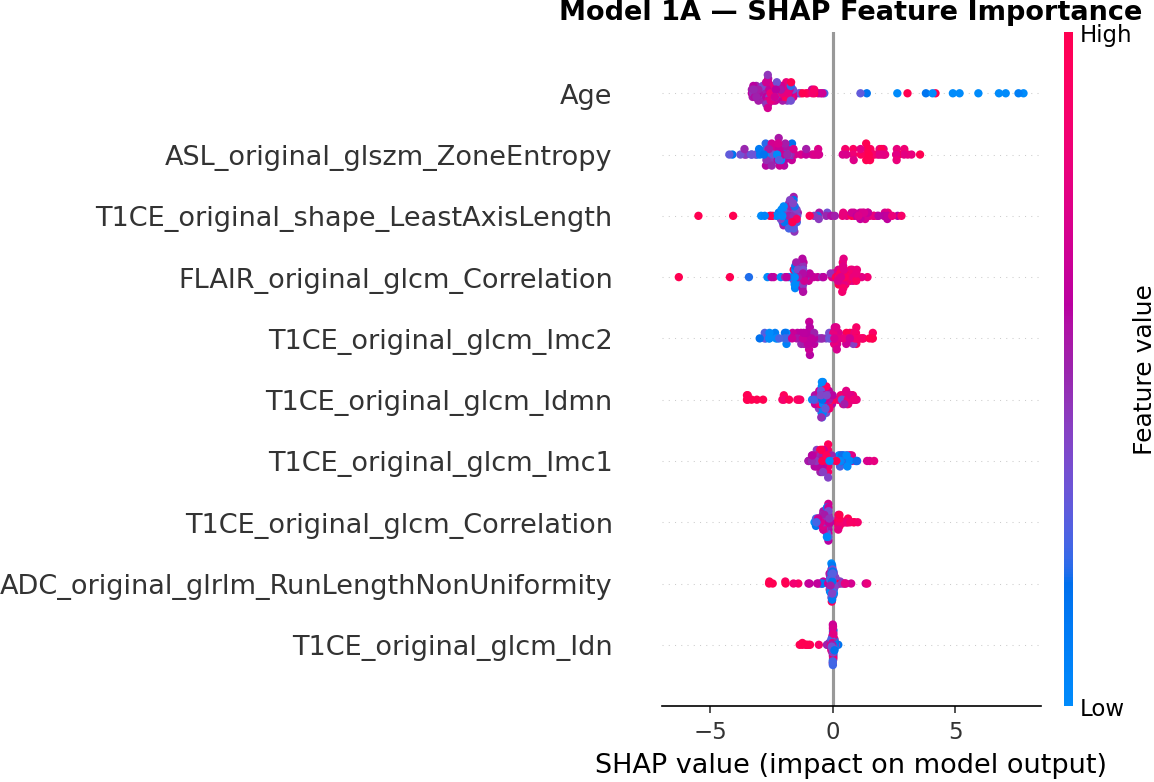
SHAP feature importance - model 1A.

To determine whether physiological sequences encoding tumor cellularity and perfusion provide additional discriminative value, we incorporated ADC and ASL-CBF features into the radiomic pipeline. Feature extraction yielded approximately 535 features prior to filtering. At a correlation threshold of 0.98 and k = 10, Gradient Boosting achieved the highest AUC of 0.753 (95% CI: 0.640–0.848). At the Youden-derived threshold of 0.003, the model demonstrated high sensitivity (0.947; 95% CI: 0.542–1.000) and a PR-AUC of 0.364, indicating improved sensitivity and PR-AUC for the minority ATRX-loss class, albeit at reduced specificity. SHAP analysis demonstrated that Age remained the dominant predictor, while ASL-derived texture features — particularly ASL_original_glszm_ZoneEntropy — emerged as the second most influential feature, confirming the added discriminative value of perfusion imaging in predicting ATRX mutation status (**Figure 5**).

### Model 1B: structural MRI + ADC (T1CE + FLAIR + T2 + ADC)

**Figure 6:**
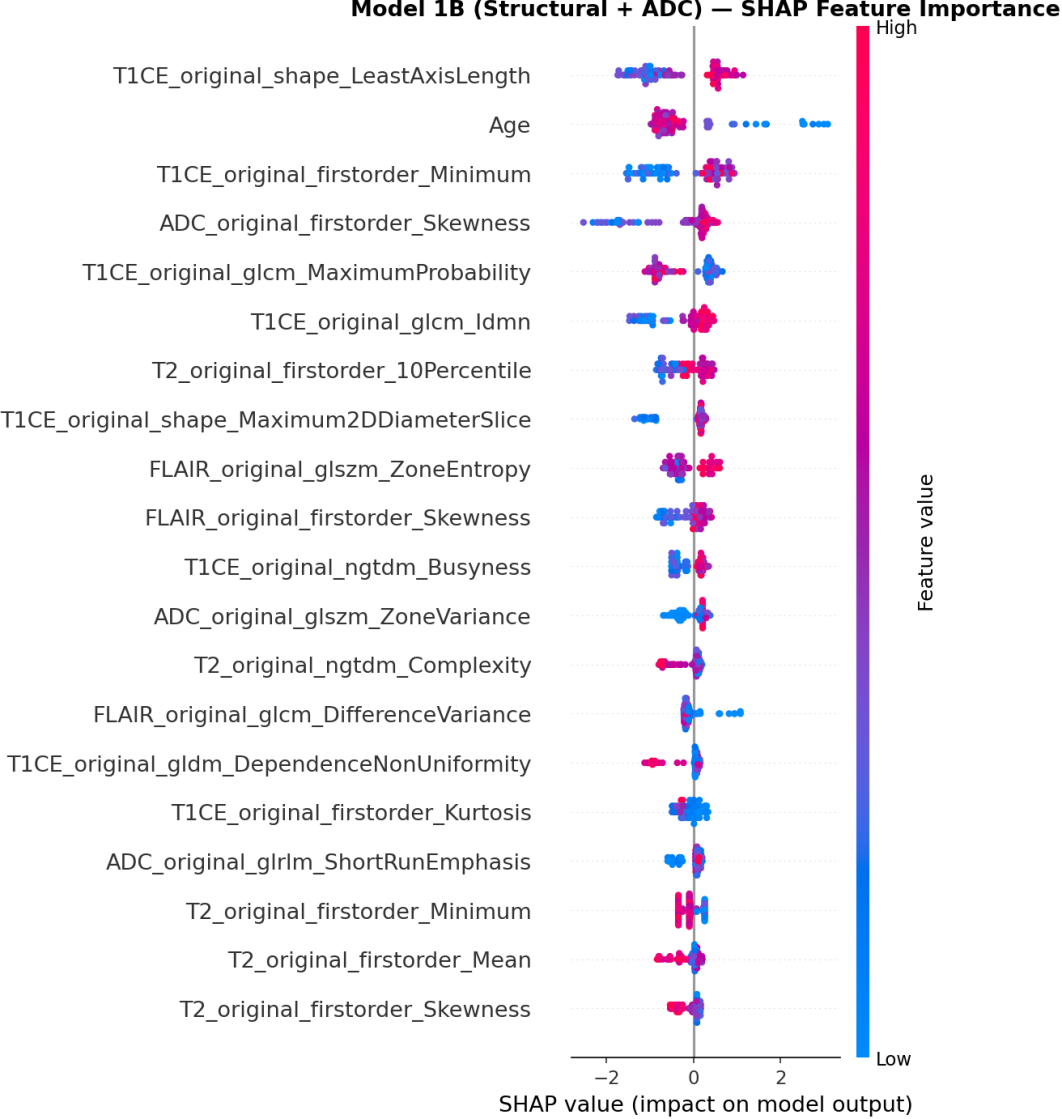
SHAP feature importance - Model 1B.

To isolate the contribution of diffusion-weighted features, Model 1B incorporated only radiomic features from structural MRI sequences alongside with ADC features. Feature extraction yielded approximately 428 features prior to filtering. At a correlation threshold of 0.97 and k = 200, LightGBM achieved the highest AUC of 0.701 (95% CI: 0.572–0.815). At the Youden-derived threshold, the model demonstrated a sensitivity of 0.842 (95% CI: 0.556–1.000), specificity of 0.586 (95% CI: 0.500–0.844), NPV of 0.944 (95% CI: 0.878–1.000), and a PR-AUC of 0.306 (95% CI: 0.183–0.512). SHAP analysis revealed that T1CE_original_shape_LeastAxisLength was the most influential predictor, followed by Age and T1CE_original_firstorder_Minimum, indicating that tumour geometry and contrast-enhancing signal intensity remain the dominant drivers even in the presence of ADC features. Notably, ADC_original_firstorder_Skewness emerged as the fourth most influential feature, reflecting asymmetry in the diffusion coefficient distribution within the tumour — a marker of microstructural heterogeneity — while ADC_original_glszm_ZoneVariance contributed further, capturing spatial variability in diffusion-related zone sizes. Despite the inclusion of ADC features, the model did not outperform the structural baseline (AUC = 0.721), suggesting that diffusion-derived radiomic features in isolation provide limited additional discriminative signal for ATRX prediction at this sample size (**Figure 6**).

### Model 1C: structural MRI + ASL (T1CE + FLAIR + T2 + ASL)

**Figure 7:**
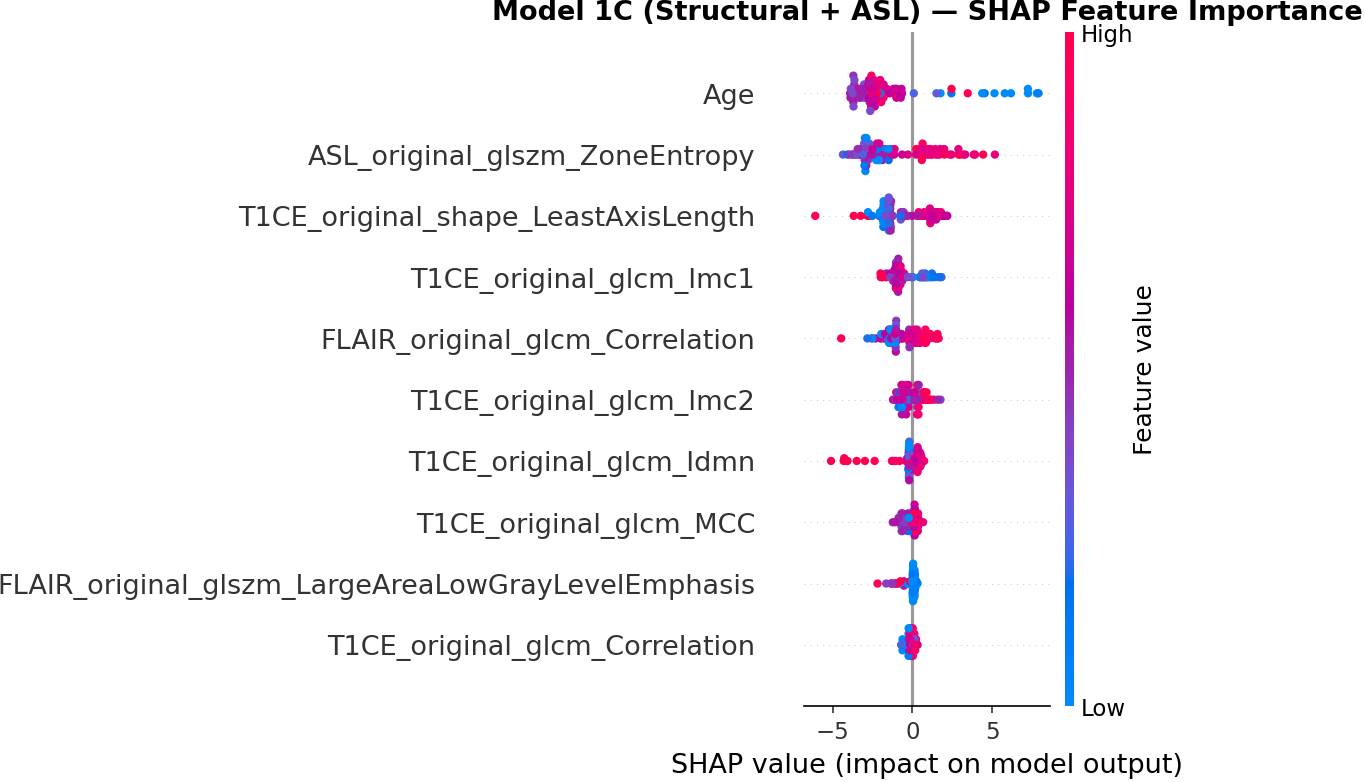
SHAP feature importance - model 1C.

To isolate the contribution of perfusion-derived features, Model 1C incorporated radiomic features from structural MRI sequences alongside ASL-CBF features only. Feature extraction yielded approximately 428 features prior to filtering. At a correlation threshold of 0.97 and k = 10, Gradient Boosting achieved the highest AUC of 0.729 (95% CI: 0.607–0.837). At the Youden-derived threshold, the model demonstrated a sensitivity of 0.684 (95% CI: 0.481–1.000), specificity of 0.736 (95% CI: 0.299–0.918), NPV of 0.914 (95% CI: 0.861–1.000), and a PR-AUC of 0.352 (95% CI: 0.210–0.580). SHAP analysis demonstrated that Age remained the dominant predictor, consistent with its role across all model configurations. ASL_original_glszm_ZoneEntropy emerged as the second most influential feature — the same ASL-derived perfusion texture feature identified in Model 1A — confirming that spatial heterogeneity in perfusion-related grey-level zones carries consistent and reproducible discriminative signal for ATRX prediction. Structural features including T1CE_original_shape_LeastAxisLength, T1CE_original_glcm_Imc1, and FLAIR_original_glcm_Correlation contributed further, reflecting the continued importance of tumour morphology and signal texture. The AUC of 0.729 represents a modest improvement over the structural baseline (AUC = 0.721), with ASL-derived perfusion heterogeneity providing incremental discriminative value when combined with structural features (**Figure 7**).

### Comparison of model 1, model 1A, model 1B and model 1C

**Figure 8:**
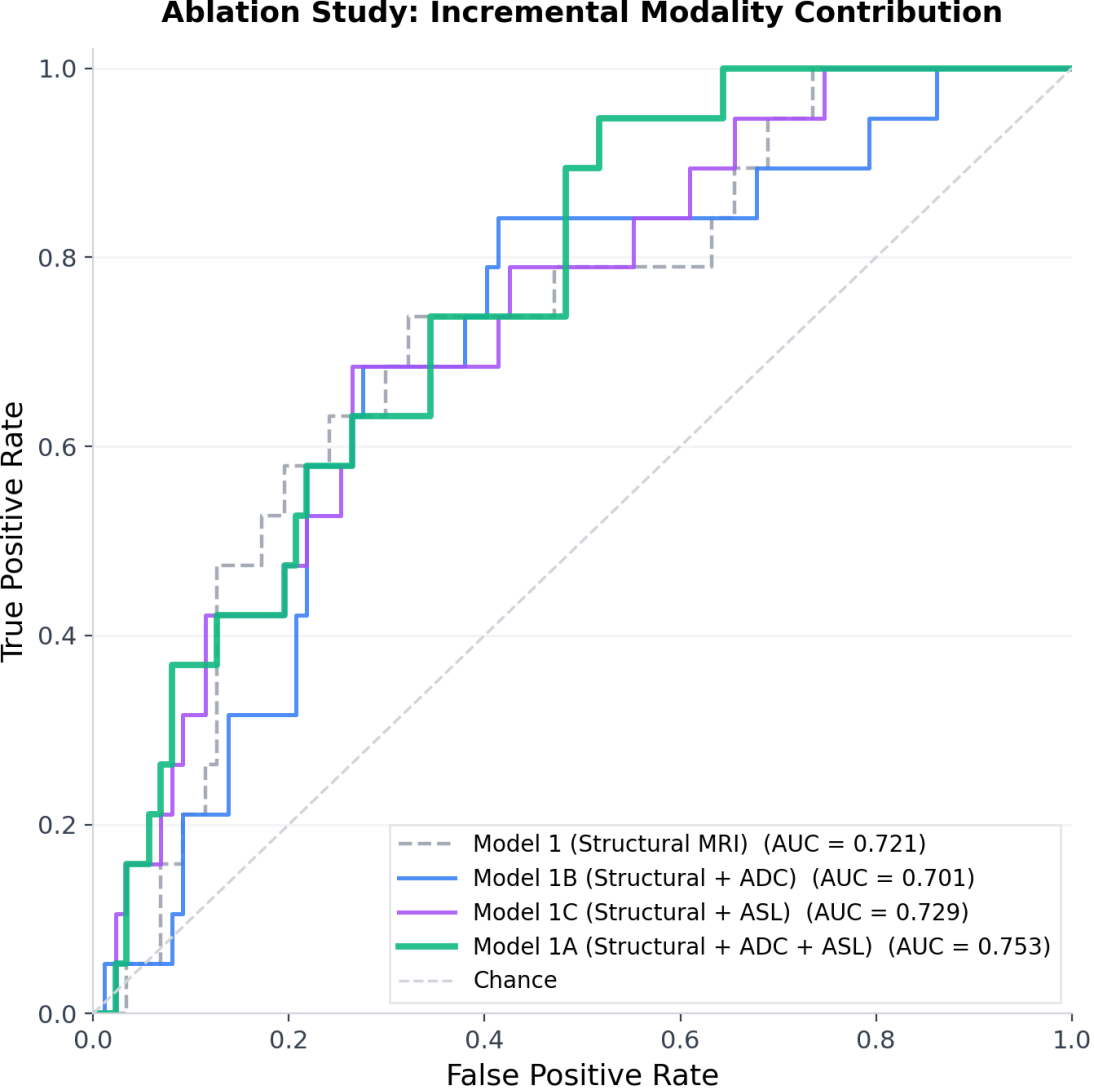
Receiver operating characteristic (ROC) curves for the ablation study comparing Model 1 (structural MRI only, AUC = 0.721), Model 1B (structural MRI + ADC, AUC = 0.701), Model 1C (structural MRI + ASL, AUC = 0.729), and Model 1A (structural MRI + ADC + ASL, AUC = 0.753).

ROC curves for all four models are presented in **Figure 8**. Model 1A consistently outperformed Model 1 across most, but not all, of the evaluated threshold and feature-count configurations. Paired DeLong tests, applied to the same 106-patient cohort, demonstrated statistically significant improvements across 11 configurations (p<0.05), with the most significant results at a correlation threshold of 0.85 and k=5, and at a threshold of 0.90 and k=5 (both p<0.001). ASL-CBF features provided consistent individual benefit over the structural baseline, with Model 1C significantly outperforming Model 1 across 13 configurations (p<0.05), including three at p<0.001. In contrast, ADC features alone did not provide reliable improvement — Model 1B showed no significant gain over Model 1 at any configuration, and at one configuration (threshold=0.90, k=20) Model 1 significantly outperformed Model 1B (p=0.007), suggesting that ADC features in isolation may introduce noise that the model cannot reliably exploit at this sample size. Model 1A significantly outperformed Model 1B across multiple configurations (p<0.05), confirming that ASL-CBF features are necessary to realise the discriminative potential of the full multiparametric model. A summary of the DeLong test results across all evaluated configurations is provided in **Table 3**. Comprehensive performance metrics for all four models with 95% confidence intervals are reported in **Table 4**. To further characterise the individual contribution of each advanced sequence, two intermediate ablation models were evaluated. Model 1B, incorporating structural MRI and ADC features, yielded an AUC of 0.701 (LightGBM, k=200), marginally below the structural-only baseline (AUC = 0.721), suggesting that ADC features in isolation may not provide a reliable additional discriminative signal at this sample size. Model 1C, incorporating structural MRI and ASL features, achieved an AUC of 0.729 (GradientBoost, k=10), representing a modest improvement over the structural baseline. The full multiparametric model (Model 1A) achieved the highest AUC of 0.753 (GradientBoost, k=10), demonstrating that ADC and ASL features carry complementary information whose combined contribution exceeds that of either modality alone (**Figure 8**).

**Table 3:** Statistically significant paired DeLong comparisons across all model configurations.

Comparison	Correlation threshold	k	AUC (model A)	AUC (model B)	ΔAUC	DeLong p-value
Model 1 vs Model 1A	0.85	5	0.508	0.726	−0.218	<0.001***
Model 1 vs Model 1A	0.90	5	0.493	0.742	−0.248	<0.001***
Model 1 vs Model 1A	0.97	10	0.526	0.743	−0.217	0.002**
Model 1 vs Model 1A	0.98	10	0.543	0.753	−0.210	0.004**
Model 1 vs Model 1A	0.99	8	0.544	0.710	−0.166	0.016*
Model 1 vs Model 1A	0.80	8	0.463	0.639	−0.175	0.016*
Model 1 vs Model 1A	0.85	16	0.447	0.629	−0.182	0.025*
Model 1 vs Model 1A	0.80	5	0.592	0.726	−0.134	0.027*
Model 1 vs Model 1B	0.90	20	0.579	0.440	+0.139	0.007**
Model 1 vs Model 1C	0.80	10	0.460	0.667	−0.207	<0.001***
Model 1 vs Model 1C	0.80	8	0.463	0.641	−0.177	0.001**
Model 1 vs Model 1C	0.90	5	0.493	0.667	−0.174	0.002**
Model 1 vs Model 1C	0.85	5	0.508	0.652	−0.143	0.003**
Model 1 vs Model 1C	0.97	10	0.526	0.729	−0.203	0.004**
Model 1 vs Model 1C	0.98	10	0.543	0.722	−0.179	0.010*
Model 1 vs Model 1C	0.95	10	0.520	0.719	−0.199	0.011*
Model 1 vs Model 1C	0.80	5	0.592	0.729	−0.137	0.017*
Model 1B vs Model 1A	0.80	80	0.455	0.652	−0.197	0.002**
Model 1B vs Model 1A	0.98	10	0.578	0.753	−0.175	0.006**
Model 1B vs Model 1A	0.97	10	0.575	0.743	−0.168	0.009**
Model 1B vs Model 1A	0.85	8	0.494	0.647	−0.153	0.009**
Model 1B vs Model 1A	0.85	32	0.493	0.673	−0.179	0.014*
Model 1B vs Model 1A	0.90	5	0.593	0.742	−0.149	0.024*
Model 1B vs Model 1A	0.80	5	0.593	0.726	−0.133	0.028*
Model 1C vs Model 1A	0.97	20	0.688	0.530	+0.159	0.003**
Model 1C vs Model 1A	0.90	5	0.667	0.742	−0.074	0.021*
Model 1C vs Model 1A	0.85	5	0.652	0.726	−0.074	0.021*
Model 1C vs Model 1A	0.85	32	0.549	0.673	−0.124	0.031*

Model 1 included radiomic features from T1CE, T2, and FLAIR. Model 1B additionally incorporated ADC features only. Model 1C additionally incorporated ASL-CBF features only. Model 1A incorporated both ADC and ASL-CBF features. ΔAUC was calculated as AUC (Model B) minus AUC (Model A); negative values indicate that Model B outperforms Model A. Two comparisons with positive ΔAUC (Model 1 vs Model 1B at thresh=0.90, k=20; Model 1C vs Model 1A at thresh=0.97, k=20) reflect configurations where the simpler model outperformed, consistent with the overall finding that ADC features alone do not reliably improve discrimination at this sample size. Only statistically significant paired DeLong comparisons are shown (p<0.05). *p<0.05, **p<0.01, ***p<0.001.

**Table 4:** Performance metrics with 95% confidence intervals.

Metric	Model 1	Model 1A	Model 1B	Model 1C
ROC-AUC	0.721 (95% CI: 0.588–0.833)	0.753 (95% CI: 0.639–0.855)	0.701 (95% CI: 0.572–0.815)	0.729 (95% CI: 0.607–0.837)
PR-AUC	0.322 (95% CI: 0.193–0.536)	0.364 (95% CI: 0.219–0.601)	0.306 (95% CI: 0.183–0.512)	0.352 (95% CI: 0.210–0.580)
Balanced Accuracy	0.708 (95% CI: 0.629–0.832)	0.715 (95% CI: 0.664–0.827)	0.714 (95% CI: 0.629–0.832)	0.710 (95% CI: 0.628–0.829)
Sensitivity	0.737 (95% CI: 0.500–1.000)	0.947 (95% CI: 0.529–1.000)	0.842 (95% CI: 0.556–1.000)	0.684 (95% CI: 0.481–1.000)
Specificity	0.678 (95% CI: 0.284–0.905)	0.483 (95% CI: 0.370–0.924)	0.586 (95% CI: 0.500–0.844)	0.736 (95% CI: 0.299–0.918)
NPV	0.922 (95% CI: 0.860–1.000)	0.977 (95% CI: 0.886–1.000)	0.944 (95% CI: 0.878–1.000)	0.914 (95% CI: 0.861–1.000)

Model 1 included radiomic features from structural MRI sequences only: T1CE, T2, and FLAIR. Model 1A additionally incorporated physiologic MRI features from ADC and ASL-CBF. ROC-AUC and PR-AUC are threshold-independent metrics; balanced accuracy, sensitivity, specificity, and NPV were calculated at the Youden-derived operating threshold. Model 1B incorporated structural MRI and ADC features only. Model 1C incorporated structural MRI and ASL-CBF features only. PR-AUC was included because of class imbalance in ATRX-loss cases. 95% confidence intervals were estimated using 1, 000 bootstrap iterations. ROC-AUC = receiver operating characteristic area under the curve; PR-AUC = precision-recall area under the curve; NPV = negative predictive value; CI = confidence interval.

### Precision-recall analysis

Given the class imbalance in ATRX mutation status, we additionally evaluated all four models using precision-recall curves. Model 1A achieved the highest PR-AUC of 0.364, followed by Model 1C (0.352), Model 1 (0.322), and Model 1B (0.306). All four models performed substantially above the baseline prevalence of 0.18, confirming meaningful discriminative performance for the minority ATRX-loss class beyond chance across all configurations. The incremental improvement in PR-AUC from Model 1 to Model 1A further supports the added value of combining ADC and ASL-CBF features for minority-class detection.

## Discussion

### Principal findings

Our study suggests that adding ADC and ASL features may provide modest incremental value for ATRX prediction beyond structural MRI and clinical variables. Model 1A achieved a higher ROC-AUC than the structural MRI model (0.721 vs 0.753, p<0.05), improved PR-AUC for the minority ATRX-loss class, and demonstrated higher sensitivity. Across classifiers, the addition of ADC and ASL improved discrimination in five of six machine learning models, supporting our hypothesis that diffusion- and perfusion-derived radiomic features provide complementary information beyond structural MRI and clinical variables. SHAP analysis further showed that age remained the dominant predictor, while ASL-derived texture features emerged among the most influential imaging features in Model 1A. The ablation analysis offered additional sequence-level context. ASL-CBF showed a more consistent contribution than ADC when evaluated separately, while ADC alone did not outperform the structural baseline. However, the full Model 1A achieved the highest ROC-AUC and PR-AUC, indicating that ADC and ASL may offer complementary physiological insights when combined. Although adding ADC and ASL-CBF features significantly improved discrimination, the actual benefit was modest, with ROC-AUC increasing from 0.721 to 0.753 and PR-AUC from 0.322 to 0.364. Furthermore, the increased sensitivity of model 1A came at the cost of specificity, suggesting a sensitivity-focused operating point rather than a broadly superior, clinically useful model. These results suggest that incorporating advanced physiologic sequences like ADC and ASL offers only incremental value for ATRX prediction in GBM.

### Biological interpretation and explainability

The improvement in diagnostic performance with the addition of ADC and ASL is biologically coherent and plausible in ATRX-altered GBM. ATRX encodes a chromatin-remodeling protein involved in replication stress, telomere maintenance, and DNA repair, making it an essential factor in genome stability (5, 24). These mechanisms may influence tumor growth, microstructural organization, necrosis, vascular adaptation, and treatment-related vulnerability (7). Yu et al. (2025) demonstrated that ATRX-loss tumors, in addition to the enrichment of DNA response pathways, showed alterations in the RTK-RAS pathway and, in a differential expression analysis, exhibited differences in cytokines, chemokine activity, extracellular collagen matrix, and stromal components in GBM (25). Therefore, relying solely on conventional morphology may not fully capture ATRX-related imaging phenotypes. ADC-derived radiomic features reflect microstructural heterogeneity related to diffusion, whereas ASL-CBF features capture perfusion and vascular heterogeneity (26, 27). Our model identified ASL-derived zone entropy, indicating voxels with similar perfusion. High entropy suggests fewer voxels share the same perfusion within a tumor area, underscoring tumor heterogeneity as a key predictor. This also emphasizes the role of perfusion heterogeneity in the tumor-encoded ATRX status.

SHAP explainability showed that the models relied on different predictive signals across configurations. The structural MRI model was driven mainly by age and T1CE-, T2-, and FLAIR-derived shape, intensity, and texture features, suggesting that it inferred ATRX status from macroscopic tumor phenotype, including enhancing tumor geometry and signal heterogeneity. In contrast, the extended multiparametric model shifted toward a physiologic attribution pattern, with age remaining the dominant clinical predictor; ASL-derived texture emerged as the most influential imaging feature, while the ADC-derived texture feature also contributed to prediction. Because ASL features reflect spatial heterogeneity in perfusion-related gray-level zones, and ADC texture features reflect heterogeneity in diffusion-related texture patterns, these findings suggest that ATRX-relevant information may be encoded in both vascular and microstructural tumor heterogeneity. This shift supports the central hypothesis that ADC and ASL add biologically meaningful information beyond structural MRI by capturing functional heterogeneity related to perfusion organization and diffusion-related microstructure.

### Comparison with previous studies

Our findings are consistent with prior literature supporting the prediction of ATRX status using radiomics-based machine learning, although this remains more challenging, with pooled sensitivities of 0.68 and a pooled AUC of 0.84, compared with predicting other tumor biomarkers, such as IDH (13). Wu et al. (2022) developed a nomogram to predict ATRX loss, reporting that age, sex, and radiomic features from structural MRI (T1, T2, FLAIR) and functional MRI (ADC, DWI, ASL) sequences are the most influential factors in determining ATRX status in low-grade gliomas. The model achieved a validation c-index of 0.84 and demonstrated good calibration (21). This aligns with our findings, which identified age as the most important predictive factor in all four models. Additionally, Sohn et al. (2021) developed two classifiers, binary relevance and an ensemble trained on T1 contrast and T2 radiomics features, to predict multiple genetic biomarkers in GBM and IDH-mutant astrocytomas. Specifically, for ATRX prediction, the classifiers yield AUCs of 0.82 and 0.77, respectively (28). Although prior studies have reported higher AUCs for ATRX prediction, our results fall toward the lower end of the published range. However, direct comparison is difficult because prior studies vary substantially in tumor grade, cohort composition, MRI sequences, modeling strategies, and validation designs, and many include lower-grade or mixed-grade glioma cohorts rather than GBM alone (29, 30). Our study focused specifically on GBM, in which ATRX loss is uncommon, and imaging heterogeneity is substantial. Therefore, the modest performance may be due to a more challenging GBM-only prediction task and class imbalance. Notably, although overall discrimination was modest, the extended model showed high sensitivity (0.947) but at the expense of lower specificity (0.483) at the Youden-based threshold. This indicates that ADC and ASL-CBF could help identify additional ATRX-loss cases.

### Clinical implications

Clinically, our findings should be considered hypothesis-generating and complementary, not definitive for diagnosis. Preoperative ATRX prediction may aid in early molecular suspicion before histopathology, especially during tumor board discussions, surgical planning, and case selection for targeted molecular testing. The relevance of ASL-CBF stems from its connection to perfusion MRI, which is already employed in GBM assessment, including evaluation of treatment response and pseudoresponse after anti-VEGF therapy. Bevacizumab, which targets vascular pathways, is approved for recurrent GBM in several countries, though it has not demonstrated an overall survival benefit (31, 32). Given the reported associations between ATRX loss, DNA damage repair dysfunction, and sensitivity to radiotherapy and temozolomide, noninvasive ATRX prediction may provide treatment-relevant biological insight (7). In this context, the association between ASL-derived texture and ATRX prediction supports further investigation of perfusion heterogeneity as a potential radiogenomic marker. However, given the modest performance of the current model, these findings remain hypothesis-generating and are not sufficient for direct clinical decision-making.

### Strengths and limitations

Our study offers several strengths. First, we employed a focused, advanced MRI-sequence improvement design by directly comparing a structural MRI model with an extended multiparametric model that includes ADC and ASL-CBF within the same GBM cohort. Second, we incorporated age and sex as clinical variables, enabling evaluation of the imaging models beyond basic clinical predictors. Third, we also addressed class imbalance by using metrics such as PR-AUC, balanced accuracy, sensitivity, specificity, and bootstrapped confidence intervals, rather than relying solely on ROC-AUC. Moreover, we used paired DeLong tests to formally assess whether discrimination improved with the addition of advanced sequences. Lastly, SHAP explainability was used to identify key clinical and imaging features that influence prediction, linking performance to biologically plausible heterogeneity in diffusion and perfusion.

The study has several limitations. First, the relatively small cohort size (n=106, with 19 ATRX-loss cases) represents the primary limitation of this study and may constrain the generalizability of the findings. Although multiple strategies were employed to mitigate overfitting — including stratified cross-validation, within-fold SMOTE oversampling, class-weighted classifiers, and feature selection — external validation in a larger, independent, and ideally multi-center cohort is necessary to confirm the robustness and clinical applicability of the proposed models. Second, external validation was unavailable, limiting confidence in the reproducibility and clinical applicability of the proposed models across different institutions, scanners, MRI acquisition protocols, and patient populations. Although cross-validation provides an internal estimate of model performance, it cannot substitute for independent external validation. Therefore, the present findings should be interpreted as preliminary and require confirmation in larger, multi-institutional cohorts before clinical translation. Third, although SHAP improves interpretability, it does not establish causality between ADC/ASL texture features and ATRX-related biology.

### Future directions

Future research should confirm these results using larger, multi-center GBM cohorts with standardized MRI protocols and external validation. The present ablation analysis, which evaluated ADC-only (Model 1B) and ASL-only (Model 1C) configurations, suggests that perfusion heterogeneity captured by ASL contributes more than ADC individually, while their combination yields the strongest performance; future work should confirm whether this pattern holds in larger, independent cohorts. Since ATRX loss is associated with DNA repair deficiencies and may increase sensitivity to temozolomide and radiotherapy, MRI signatures associated with ATRX could provide important biological insights for treatment. Additionally, exploring these methods in pediatric and adolescent high-grade gliomas, especially diffuse hemispheric gliomas and H3 G34-mutant cases, might be valuable, as ATRX loss is significant in these cases and non-contrast techniques like ASL are beneficial because they eliminate the need for gadolinium (33–35).

This study explored the additive value of ADC and ASL-CBF radiomics over structural MRI sequences for ATRX prediction in GBM. Adding physiologic MRI features improved discrimination over structural MRI and clinical data, with Model 1A performing better on ROC-AUC, PR-AUC, sensitivity, and NPV than Model 1. While overall performance was modest, paired comparisons showed ADC and ASL-CBF offer complementary information beyond conventional imaging. SHAP analysis revealed that the models relied on different signals: the structural model used age and MRI features, whereas the extended model incorporated physiologic texture, particularly ASL perfusion heterogeneity and ADC microstructure. These findings suggest ATRX information may be partly encoded in vascular and diffusion heterogeneity. The current model is not ready for clinical use, but the findings support further investigation of physiologic MRI as a complementary component of noninvasive ATRX radiogenomics in GBM. Future work should validate these findings, including the ablation results from Models 1B and 1C, in larger multi-institutional GBM cohorts with standardized MRI protocols.

## Data Availability

Publicly available datasets were analyzed in this study. This data can be found here: The imaging and clinical data used in this study are publicly available from The Cancer Imaging Archive (TCIA) in the UCSD-PTGBM collection: https://www.cancerimagingarchive.net/collection/ucsd-ptgbm/.
